# Postoperative neutrophil-lymphocyte ratio predicts unfavorable outcome of acute ischemic stroke patients who achieve complete reperfusion after thrombectomy

**DOI:** 10.3389/fimmu.2022.963111

**Published:** 2022-10-07

**Authors:** Yao Feng, Xuesong Bai, Wei Li, Wenbo Cao, Xin Xu, Fan Yu, Zhaolin Fu, Qiuyue Tian, Xiaofan Guo, Tao Wang, Arman Sha, Yanfei Chen, Peng Gao, Yabing Wang, Jian Chen, Yan Ma, Fei Chen, Adam A. Dmytriw, Robert W. Regenhardt, Jie Lu, Qingfeng Ma, Bin Yang, Liqun Jiao

**Affiliations:** ^1^ Department of Neurosurgery, Xuanwu Hospital, Capital Medical University, Beijing, China; ^2^ China International Neuroscience Institute (China-INI), Beijing, China; ^3^ Department of Neurosurgery, Liaocheng Brain Hospital, Liaocheng, China; ^4^ Department of Radiology and Nuclear Medicine, Xuanwu Hospital, Capital Medical University, Beijing, China; ^5^ Beijing Key Laboratory of Magnetic Resonance Imaging and Brain Informatics, Beijing, China; ^6^ Beijing Key Laboratory of Clinical Epidemiology, School of Public Health, Capital Medical University, Beijing, China; ^7^ Department of Neurology, Loma Linda University Health, Loma Linda, CA, United States; ^8^ Department of Interventional Neuroradiology, Xuanwu Hospital, Capital Medical University, Beijing, China; ^9^ Department of Neurology, Xuanwu Hospital, Capital Medical University, Beijing, China; ^10^ Neuroendovascular Program, Massachusetts General Hospital, Harvard Medical School, Boston, MA, United States

**Keywords:** acute ischemic stroke, endovascular treatment, NLR, inflammation, prognosis, reperfusion

## Abstract

**Purpose:**

Only approximately half of anterior circulation large vessel occlusion (LVO) patients receiving endovascular treatment (EVT) have a favorable outcome. The aim of this study was to explore the association of dynamic inflammatory markers (i.e., neutrophil to lymphocyte ratios, NLR, measured at different times after EVT) as well as other potential influencing factors with unfavorable outcome among acute ischemic stroke (AIS) patients who achieved complete reperfusion after EVT.

**Methods:**

Patients treated with EVT for LVO between January 2019 to December 2021 were prospectively enrolled. Complete reperfusion was defined as modified thrombolysis in cerebral infarction (mTICI) grade 3. A modified Rankin scale at 90 days (mRS90) of 3–6 was defined as unfavorable outcome (i.e., futile reperfusion). A logistic regression analysis was performed with unfavorable outcome as a dependent variable. The receiver operating characteristic (ROC) curve and the area under the curve (AUC) were then used to determine the diagnostic values of NLR and other relevant factors.

**Results:**

170 patients with complete reperfusion (mTICI 3) were included in this study. Unfavorable outcome was observed in 70 (41.2%). Higher NLR within 24h (p=0.017) and at 3-7d (p=0.008) after EVT were an independent risk factors for unfavorable outcome at 3 months. In addition, older age, higher NIHSS scores, poor collaterals, and general anesthesia were independent predictors of unfavorable outcomes. When accounting for NLR, the diagnostic efficiency improved compared to conventional characteristics.

**Conclusion:**

Our findings suggest that advanced age, increased stroke severity, poor collaterals, general anesthesia, and NLR are independent predictors for an unfavorable clinical outcome following complete reperfusion after EVT. Neuroinflammation may merit particular attention in future studies.

## Introduction

Endovascular treatment (EVT) has proven to be effective in improving the outcome of patients with acute ischemic stroke (AIS) due to large vessel occlusion (LVO) within the anterior circulation (1). The benefits of EVT has been largely attributed to the higher rates of successful revascularization compared with thrombolysis therapy (1). The modified thrombolysis in cerebral infarction (mTICI) score of 2b and above has traditionally been considered successful reperfusion (2). Recent clinical trials have shown that EVT achieves TICI 2b-3 reperfusion in more than 85% of AIS patients (3–5). Even when achieving successful reperfusion, AIS patients with unfavorable outcome are reported in up to 48.7% (6). Thus, identification of predictors of futile reperfusion have been hotly debated in recent years. Prompt identification of patients at increased risk of adverse outcome such as futile reperfusion, cerebral infarction volumes, ischemic reperfusion (I/R) injury and/or neuroinflammation may help target patients who deserve close attention and timely treatments (7).

In previous studies, some risk factors, such as higher initial National Institute of Health Stroke Scale (NIHSS) score, older age, longer time from onset to treatment, and infarct growth despite successful reperfusion, were reported to be associated with futile reperfusion after EVT (8–11). However, these studies largely focused on successful reperfusion (mTICI 2b-3), rather than complete reperfusion (mTICI 3). A recent systematic review and meta-analysis indicates mTICI 3 is associated with superior outcome and better safety profiles than mTICI 2b (12). Therefore, identifying predictors of futile complete reperfusion could yield crucial value to optimize patient management or inform future research endeavors exploring novel adjunct therapies. Previously, Van Horn et al. (13) proposed clinical and image indicators of poor clinical outcome despite TICI 3 reperfusion. However, they did not observe serological or other postoperative predictors, which may provide further valuable information.

Inflammatory and immune responses play key roles in ischemic stroke pathophysiology, treatment effects, and outcomes. The neutrophil to lymphocyte ratio (NLR) integrates information about both nonspecific neutrophil-driven inflammation and more targeted lymphocyte immune regulation (14). Emerging evidence indicates that post-stroke immune responses can affect the neurovascular interface, causing reperfusion injury and symptomatic intracranial hemorrhage (15). Compared with previously reported serologic biomarkers, such as matrix metalloproteinase-9, tenascin-C, and thioredoxin (16, 17), NLR is easily accessible and routinely examined in clinical practice. Thus, it may serve as a more practical tool to predict outcomes for AIS patients. A recent meta-analysis analyzed NLR values for admission and post-operation separately and found that NLR was closely related to the prognosis of AIS patients (18). Previous studies have shown that NLR is a predictor of functional outcome in patients with AIS and occurrence of intracranial hemorrhage (5, 19–22), but the role of dynamic NLR in predicting outcomes for patients who achieve complete reperfusion after EVT is unknown. The aim of this study was to explore the association of dynamic inflammatory markers (i.e., NLR measured at different times after EVT) as well as other potential influencing factors with unfavorable outcome among AIS patients who achieved complete reperfusion after EVT.

## Materials and methods

### Patient population

In this retrospective single-center study, we evaluated 443 consecutive patients who underwent EVT for acute LVO within the anterior circulation from a prospectively collected database from January 2019 to December 2021. All patients were treated according to the latest clinical guidelines in a “real-world” setting.

**Figure 1:**
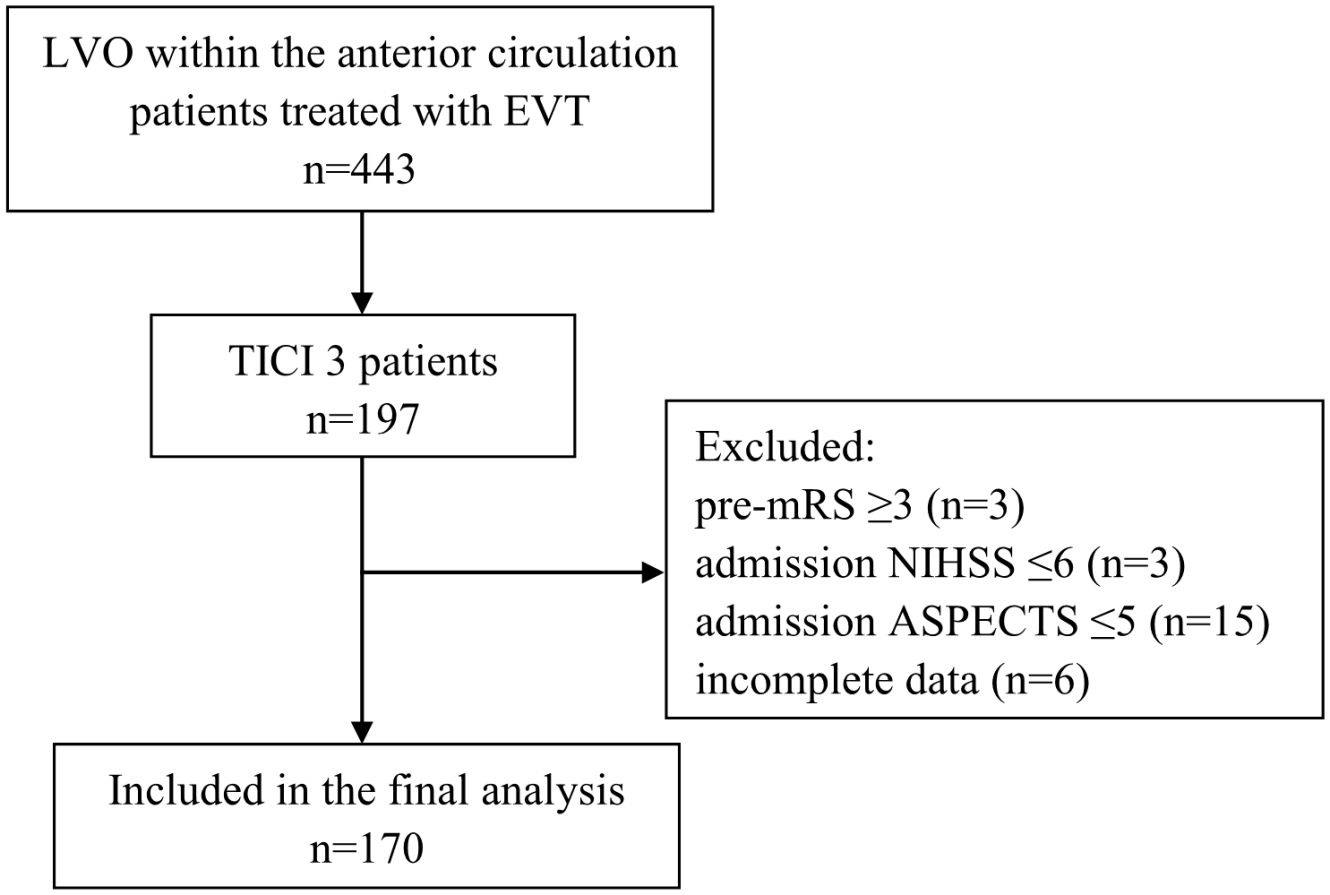
Flow chart demonstrating the number (n) of patients included in the analysis. ASPECTS, Alberta Stroke Program Early CT Score; LVO, large vessel occlusion; mRS, modified Rankin scale; EVT, endovascular treatment; NIHSS, National Institutes of Health Stroke Scale; TICI, Thrombolysis in Cerebral Infarction.

The inclusion criteria of the present study were as follows: (1) age ≥18 years; (2) AIS due to LVO within the anterior circulation; (3) complete reperfusion (defined as mTICI 3); (4) known admission National Institutes of Health Stroke Scale (NIHSS) and ASPECTS; (5) known mRS90 days after EVT The exclusion criteria were as follows: (1) pre-mRS ≥3; (2) NIHSS ≤5; (3) ASPECTS ≤5; (4) incomplete data. **Figure 1** shows the flow chart of the patient selection process. This study was approved by the institutional review board, and the need for written informed consent was waived based on the study’s retrospective design, de-identified data, and minimal patient risk.

### Baseline characteristics and image analysis

Patient demographics and clinical data were assessed, including age, sex, comorbidities (hypertension [HTN], diabetes mellitus [DM], coronary artery disease [CAD], etc.), presentation NIHSS, stroke type according to the Trial of Org 10172 in Acute Stroke Treatment classification (TOAST), intravenous thrombolysis, anesthesia type, time from onset-to-reperfusion (OTR), etc. Routine blood tests including glucose level and lipid panel (triglyceride [TG], total cholesterol [TC], high-density lipoprotein [HDL], low-density lipoprotein [LDL]) were measured at admission. Neutrophil and lymphocyte levels were collected at admission (neutrophil1, lymphocyte1 and NLR1), within 24h (neutrophil2, lymphocyte2 and NLR2) and at 3-7d (neutrophil3, lymphocyte3 and NLR3) after EVT (7).

Imaging data included presentation ASPECTS from non-contrast computed tomography (NCCT) and occlusion site from initial CTA and confirmed with digital subtraction angiography (DSA). Collateral status was evaluated using the American Society of Interventional and Therapeutic Neuroradiology/Society of Interventional Radiology (ASITN/SIR) collateral grading system and collateral status was dichotomized into poor (ASITN/SIR 0–2) or good (ASITN/SIR 3–4) (23). Reperfusion status was evaluated using the modified Thrombolysis in Cerebral Infarction (mTICI) score. CT images were analyzed by an experienced radiologist (>5 years of experience) and DSA images were analyzed by an experienced neuroradiologist (>10 years of experience).The angiographic result was assessed on the final DSA image series and was classified according to the mTICI scale; complete reperfusion was defined as mTICI 3.

### Statistical analysis

Participants were classified into two groups by 90-day mRS (mRS90): mRS90 0-2 vs 3-6 (13). Continuous variables are presented as median (interquartile range [IQR]) due to their skewed distribution. Categorical variables are presented as frequency and percentage. The non-parametric Wilcoxon test was used to compare group differences for continuous variables, and chi-square test or Fisher exact test was used for categorical variables as appropriate.

All tests were 2-tailed, and the level of significance was set at p<0.05. Multivariable analysis was performed with a logistic regression model including those factors with a *p*-value of ≤0.05. Subsequently, a backward stepwise selection was applied to identify the parsimonious model restricted to the most relevant prognostic factors; a significance level of 0.05 for a variable to stay in the model was chosen. Odds ratios (ORs) and 95% confidence intervals (CIs) were calculated for unfavorable outcome. Data were analyzed using IBM SPSS Version 23.0 software (IBM Corporation, Armonk, New York, USA). The receiver operating characteristic (ROC) curve and the area under the curve (AUC) were then used to determine the diagnostic values of these factors to predict unfavorable outcome.

## Results

There were 197/443 (44.5%) patients who underwent EVT for an anterior circulation large vessel occlusion with complete reperfusion (mTICI 3). The inclusion criteria were met by 170 patients (**Figure 1**).

The median age of the patients was 66.0 (IQR, 58.8-74.3) years and 115 (67.6%) were male. One hundred (58.8%) patients had a favorable clinical outcome at 3 months. Compared to patients with a favorable outcome, those with unfavorable outcomes were more likely to be older (p<0.001). Patients with DM (p=0.019) or CAD (p=0.015) were also more likely to have unfavorable outcomes. Patients in the unfavorable group had higher NIHSS scores (p<0.001) on admission, less collateral compensation (p<0.001), and were more likely to undergo general anesthesia (p<0.001). Regarding serology, there were significant differences in glucose (p=0.041), NLR1 (p<0.001), NLR2 (p<0.001), and NLR3 (p<0.001). However, there were no significant differences in lipid panel (p>0.05) (**Table 1**).

**Table 1:** Patient demographics, clinical characteristics, blood test and procedure information.

Characteristics	Total (n = 170)	Favorable outcome (mRS90 0-2)(n = 100)	Unfavorable outcome (mRS90 3-6)(n = 70)	P value
**Demographics**				
Age, median (IQR)	66.0 (58.8-74.3)	64.0 (56.0-70.8)	71.0 (64.8-78.0)	<0.001^a^
Male, n (%)	115 (67.6)	68 (68.0)	47 (67.1)	0.906^b^
HTN, n (%)	111 (65.3)	61 (61.0)	50 (71.4)	0.160^b^
DM, n (%)	49 (28.8)	22 (22.0)	27 (28.6)	0.019^b^
Dyslipidemia, n (%)	43 (25.3)	23 (23.0)	20 (28.6)	0.411^b^
CAD, n (%)	42 (24.7)	18 (18.0)	24 (24.3)	0.015^b^
AF, n (%)	47 (27.6)	27 (27.0)	20 (28.6)	0.822^b^
Smoking, n (%)	63 (37.1)	36 (36.0)	27 (38.6)	0.733^b^
Drinking, n (%)	58 (34.1)	36 (36.0)	22 (31.4)	0.536^b^
Stroke history, n (%)	31 (18.2)	14 (14.0)	17 (24.3)	0.087^b^
Antiplatelet use, n (%)	36 (21.2)	17 (17.0)	19 (27.1)	0.111^b^
Anticoagulation use, n (%)	15 (8.8)	10 (10.0)	5 (7.1)	0.518^b^
**Clinical characteristics**				
Intravenous thrombolysis, n (%)	62 (36.5)	37 (37.0)	25 (35.7)	0.864^b^
NIHSS, median (IQR)	15 (12-19)	13 (10-16)	18 (14-21)	<0.001^a^
ASPECT score, median (IQR)	9 (8-9)	9 (8-10)	8 (7-9)	0.242^a^
Occlusion site, n (%)				
ICA	61 (35.9)	32 (32.0)	29 (41.4)	0.189^b^
MCA-M1	87 (51.2)	57 (57.0)	30 (42.9)	
MCA-M2	22 (12.9)	11 (11.0)	11 (15.7)	
Right side, n (%)	76 (44.7)	49 (49.0)	27 (38.6)	0.211^b^
Good collaterals, n (%)	70 (41.2)	57 (57.0)	13 (18.6)	<0.001^b^
TOAST, n (%)				
LAA	66 (38.8)	44 (44.0)	22 (31.4)	0.274^c^
Cardioembolic	100 (58.8)	54 (54.0)	46 (65.7)	
Others	4 (2.4)	2 (2.0)	2 (2.9)	
**Blood test**				
Glucose, mmol/L, median (IQR)	7.28 (5.99-9.52)	6.91 (5.93-9.16)	8.11 (6.10-10.46)	0.041^a^
TG, mmol/L, median (IQR)	1.18 (0.78-1.73)	1.17 (0.78-1.67)	1.20 (0.79-1.74)	0.804
TC, mmol/L, median (IQR)	4.42 (3.71-4.92)	4.43 (3.80-4.90)	4.42 (3.59-5.00)	0.689
HDL, mmol/L, median (IQR)	1.15 (0.99-1.38)	1.14 (0.95-1.37)	1.16 (1.01-1.38)	0.358
LDL, mmol/L, median (IQR)	2.63 (2.07-3.33)	2.69 (2.26-3.40)	2.59 (1.97-3.25)	0.210
Neutrophil1, 10^9^/L, median (IQR)	6.75 (6.14-8.85)	6.01 (4.90-8.28)	7.35 (4.57-9.80)	0.184^a^
Lymphocyte1, 10^9^/L, median (IQR)	1.34 (0.93-1.84)	1.43 (1.00-1.90)	1.26 (0.74-1.73)	0.080^a^
NLR1, median (IQR)	4.95 (2.66,7.59)	4.17 (2.65-6.99)	5.85 (2.63-10.67)	0.044^a^
Neutrophil2, 10^9^/L, median (IQR)	7.74 (5.96-10.29)	7.31 (5.45-8.96)	8.57 (6.81-11.39)	0.001^a^
Lymphocyte2, 10^9^/L, median (IQR)	1.05 (0.69-1.39)	1.19 (0.85-1.47)	0.80 (0.48-1.23)	<0.001^a^
NLR2, median (IQR)	7.51 (4.60-13.26)	6.82 (4.21-9.45)	10.87 (6.39-19.00)	<0.001^a^
Neutrophil3, 10^9^/L, median (IQR)	6.23 (4.80-8.32)	5.65 (4.63-7.23)	7.53 (5.74-8.95)	<0.001^a^
Lymphocyte3, 10^9^/L, median (IQR)	1.22 (0.87-1.57)	1.34 (0.96-1.70)	1.04 (0.77-1.35)	<0.001^a^
NLR3, median (IQR)	5.25 (3.40-7.94)	4.29 (2.77-6.69)	6.39 (4.47-10.64)	<0.001^a^
**Procedure**				
General anesthesia, n (%	35 (20.6)	15 (15.0)	20 (28.6)	0.031^b^
OTD, min, median (IQR)	262.5 (150.0-384.8)	279.0 (151.0-408.0)	244.5 (148.5-351.5)	0.369^a^
PTR, min, median (IQR)	36.5 (24.8-51.0)	33.0 (23.0-48.8)	39.0 (25.8-553.5)	0.094^a^
OTR, min, median (IQR)	428.0 (330.3-574.3)	446.0 (332.5-593.0)	418.5 (322.8-553.5)	0.585^a^

^a^Analysed by Mann-Whitney U test; ^b^Analysed by Chi-square test; ^c^Analysed by Fisher’s exact test;

HTN, hypertension; DM, diabetes mellitus; AF, atrial fibrillation; CAD, coronary artery disease; NIHSS, National Institutes of Health Stroke Scale; ASPECT, Alberta Stroke Program Early CT score; ICA, internal carotid artery; MCA, middle cerebral artery; TOAST, the Trial of Org 10172 in Acute Stroke Treatment classification; LAA, large artery atherosclerosis; NLR, neutrophil to lymphocyte ratio; OTR, time of onset to recanalization; mTICI, the modified Thrombolysis in Cerebral Infarction score; IQR, interquartile rang;

Neutrophil1, Lymphocyte1 and NLR1 were measured at admission;

Neutrophil2, Lymphocyte2 and NLR2 were measured within 24h after EVT;

Neutrophil3, Lymphocyte3 and NLR3 were measured at 3-7d after EVT.

Multivariable logistic regression analysis showed that higher NLR within 24h or at 3-7d after EVT independently increased the odds of unfavorable outcome at 3 months (OR=1.070, 95% CI 1.012 to 1.131, p=0.017; OR=1.129, 95% CI 1.032 to 1.236, p=0.008). In addition, older age, higher NIHSS scores, poor collaterals, and general anesthesia were also significant determinants of poor functional outcome (**Table 2**). We calculated the diagnostic values for the conventional model alone (Model 1, including age, DM, CAD, NIHSS, good collaterals and glucose) and conventional model with NLR parameters (Model 2 [convention model plus NLR1], Model 3 [convention model plus NLR2], and Model 4 [convention model plus NLR3]). The AUCs for each model were 0.864, 0.872, 0.872 and 0.883, respectively.

**Table 2:** Independent predictors of unfavorable outcome despite complete reperfusion.

Variable	Model 1		Model 2		Model 3		Model 4	
	OR (95%CI)	P	OR (95%CI)	P	OR (95%CI)	P	OR (95%CI)	P
Age	1.059 (1.018-1.102)	0.004*	1.064 (1.021-1.108)	0.003*	1.052 (1.009-1.096)	0.017*	1.047 (1.005-1.092)	0.029*
DM	1.905 (0.654-5.549)	0.237	2.151 (0.725-6.385)	0.168	2.077 (0.691-6.241)	0.193	2.231 (0.737-6.752)	0.156
CAD	1.339 (0.483-3.713)	0.575	1.341 (0.484-3.716)	0.573	1.174 (0.408-3.379)	0.766	1.211 (0.418-3.512)	0.725
NIHSS	1.246 (1.121-1.385)	<0.001*	1.249 (1.120-1.391)	<0.001*	1.231 (1.104-1.372)	<0.001*	1.226 (1.098-1.367)	<0.001*
Good collaterals	0.161 (0.066-0.392)	<0.001*	0.166 (0.068-0.406)	<0.001*	0.158 (0.063-0.396)	<0.001*	0.135 (0.052-0.353)	<0.001*
Glucose	0.946 (0.817-1.096)	0.461	0.931 (0.801-1.082)	0.350	0.924 (0.790-1.080)	0.320	0.955 (0.822-1.110)	0.548
General anesthesia	4.507 (1.514-13.412)	0.007*	4.256 (1.423-12.733)	0.010*	4.211 (1.351-13.130)	0.013*	4.444 (1.434-13.768)	0.010*
NLR1	–	–	1.072 (0.995-1.154)	0.066	–	–	–	–
NLR2	–	–	–	–	1.070 (1.012-1.131)	0.017*	–	–
NLR3	–	–	–	–	–	–	1.129 (1.032-1.236)	0.008*

OR, odds ratio; CI, confidence interval; DM, diabetes mellitus; CAD, coronary artery disease; NIHSS, National Institutes of Health Stroke Scale; NLR, neutrophil to lymphocyte ratio

NLR1 were measured at admission;

NLR2 were measured within 24h after EVT;

NLR3 were measured at 3-7d after EVT.

*P<0.05.

## Discussion

In this study, we confirm that futile reperfusion is a common phenomenon in anterior circulation stroke patients achieving complete reperfusion, with a rate of 41.2%. This result is consistent with a previous study (13). Additionally, several variables, including older age, higher NIHSS score, poor collaterals, general anesthesia, NLR within 24h, and NLR during 3-7d after EVT were independent predictors for unfavorable clinical outcome at 3-month follow-up. When NLR is combined with a model of conventional determinants of outcome, the diagnostic efficiency is improved. Results of the current study could provide future targets to improve outcomes for patients with futile reperfusion, such as controlling the inflammatory cascade and EVT selection considerations.

The inflammatory cascade has been implicated in the ischemic process at different stages of stroke, during which brain damage and repair coexist (24, 25). Inflammatory activity is likely initiated to clear damaged tissue and promote synapse reconstruction *via* cytokines released by immune cells. However, continuous inflammatory activity beyond the acute stage may aggravate injury and hamper repair (26). Within the first hours after reperfusion, neutrophils are among the first cells to penetrate hypoxic tissue; they can cause damage to the blood brain barrier and contribute to injury of surrounding tissues (27). NLR is seen as a systemic marker of subclinical inflammation, and an increased ratio is of prognostic value in several disorders. Thus, perioperative NLR values likely reflect the state of inflammation in stroke patients.

**Figure 2:**
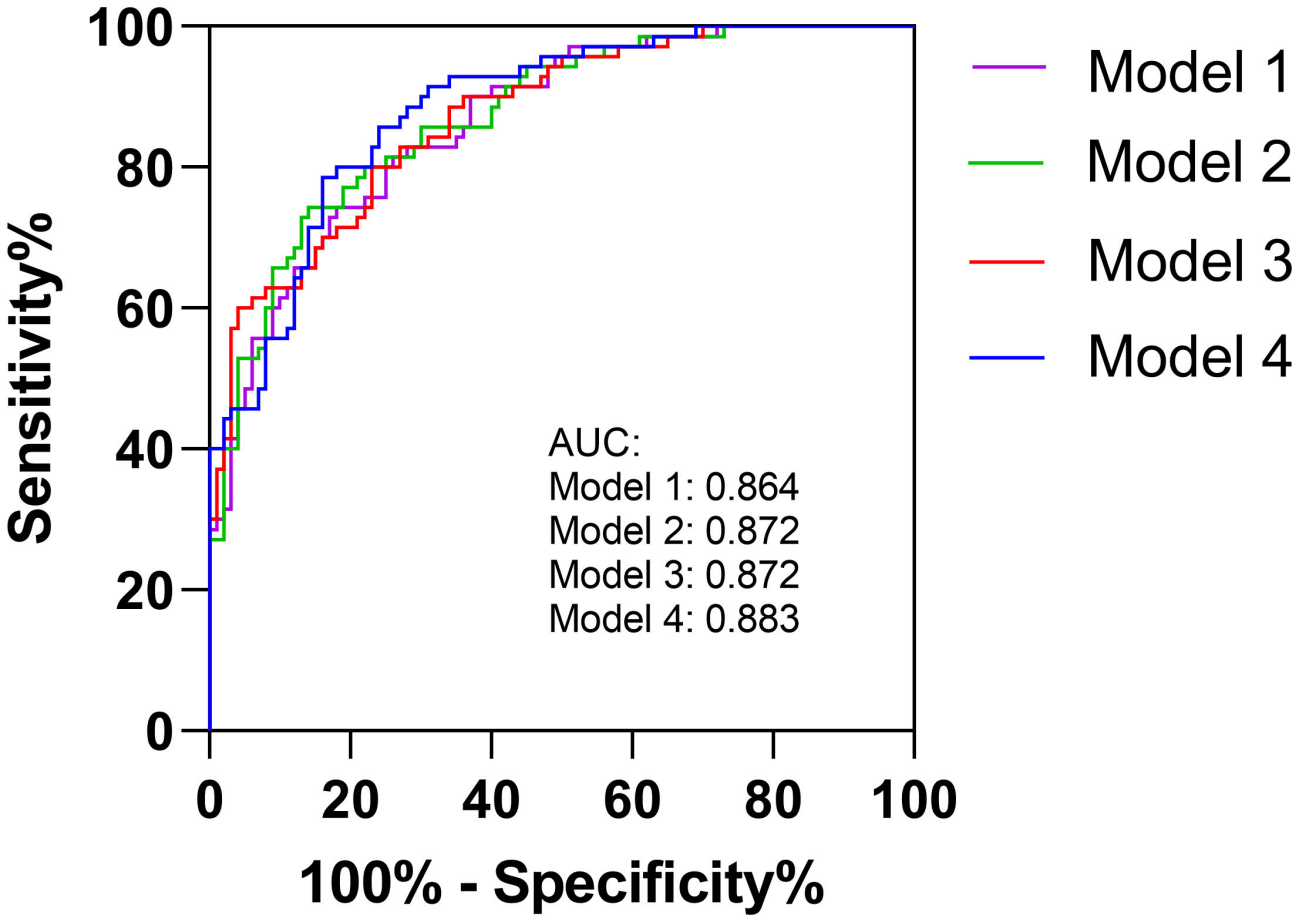
The AUCs for conventional model (Model 1) and conventional model with NLR parameters (Model 2, Model 3 and Model 4).

Even when achieving complete reperfusion (i.e., mTICI 3), a substantial proportion of AIS patients still do not achieve satisfactory outcomes. Neuroinflammation may help to explain futile reperfusion, and NLR is an easily measurable parameter in routine clinical practice. Thus, we focus on this marker to illustrate the association of inflammation with prognosis in this special group of patients. In multivariable analyses, 24 hour and 3-7 day NLR are significantly associated with outcome whereas admission NLR is not, suggesting that postoperative neuroinflammation may be strongly associated with unfavorable outcome at 90 days. Aly et al. (7) also found that lower NLR at 3–7 days rather than on admission is associated with successful reperfusion and an independent predictor of favorable clinical outcomes. Besides, Chen et al. also concluded that day 1 NLR is better than admission NLR for predicting AIS patients outcome after reperfusion therapy with a large clinical population (28). A current meta-analysis (18) also mentioned that delayed NLR (postoperative NLR) has a better prognostic utility than admission NLR because of larger standard mean difference for good functional outcomes. This could be due to underlying pathophysiology; as lymphocyte would enter into ischemic area of the brain 1-2 days after initial cerebral ischemia, and subsequently combined with other pro-inflammatory cytokine to further aggravate the damage (29). Therefore, postoperative NLR may improve prognostication for functional outcomes (15). In another study examining interleukin-6 (IL-6) as a marker of inflammation, high IL-6 levels at 24 hours were associated with futile reperfusion after adjusting for confounders (30). When NLR1, NLR2, and NLR3 were respectively added into our models for predicting outcomes, the efficiency improved compared to the model with conventional predictors alone (**Figure 2**). Therefore, postoperative neuroinflammation may be a promising and modifiable target for future research to improve outcomes for patients who may otherwise experience futile reperfusion.

In addition to NLR, some other factors, such as age, baseline NIHSS score, and collateral status also influenced final outcomes after complete reperfusion. Older age is a widely-accepted risk factor for futile reperfusion, and the benefit of EVT is known to decrease with advanced age possibly attributed to increased frailty (31). The NIHSS score closely reflects the stroke severity and has been previously associated with outcomes after EVT (32). General anesthesia is more often utilized in critically ill patients in our practice, and these patients tend to benefit less from EVT. In practices where general anesthesia is routinely used, it is unclear whether this signal would persist given data supporting its non-inferiority compared to conscious sedation in EVT (33). Lastly, collateral circulation is known to help sustain viable cerebral tissue prior to successful reperfusion (34, 35), which could explain the reason why patients with good collaterals are more likely to have a favorable outcome. These various factors can provide interventionalists with new perspectives toward preventing futile reperfusion.

There are limitations to our study, principally related to bias inherent to the study design. We also did not continuously measure serologic markers through 90 days after EVT. Intercurrent complications such as infection which is common in severe stroke leading to an increase in neutrophil and NLR cannot be ruled out.

## Conclusions

Even with technically complete reperfusion, several factors are strongly associated with unfavorable outcomes. Our findings suggest that advanced age, increased stroke severity, poor collaterals, general anesthesia, and NLR post-EVT are independent predictors of an unfavorable clinical outcome following complete reperfusion. Neuroinflammation likely merits further attention in future studies.

## Data availability statement

The original contributions presented in the study are included in the article. Further inquiries can be directed to the corresponding authors.

## Ethics statement

Ethical review and approval was not required for the study on human participants in accordance with the local legislation and institutional requirements. Written informed consent for participation was not required for this study in accordance with the national legislation and the institutional requirements.

## Author contributions

YF, XB and WL contributed to the initial idea for this study. WC, XX, and FY finished the study design. JL, PG, BY, YM, FC and LJ were consulted about the clinical issues. ZF, QT, and XG contributed to the original draft. TW, AS, AAD, RWR, QM XB, YC, YW and JC were responsible for the revision of the draft. YF and XB contributed equally to this article.

## Conflict of interest

The authors declare that the research was conducted in the absence of any commercial or financial relationships that could be construed as a potential conflict of interest.

## Publisher’s note

All claims expressed in this article are solely those of the authors and do not necessarily represent those of their affiliated organizations, or those of the publisher, the editors and the reviewers. Any product that may be evaluated in this article, or claim that may be made by its manufacturer, is not guaranteed or endorsed by the publisher.
